# Building health sciences library collections: a handbook

**DOI:** 10.29173/jchla29835

**Published:** 2025-04-01

**Authors:** Amanda Headrick

**Affiliations:** Toronto, ON, Canada

Inman M, Rose M, editors. **Building health sciences library collections: a handbook**. Lanham, MD: Rowman & Littlefield; 2023. Hardcover: 144 p. ISBN: 978-1-5381-72727-1. Price: USD$120.00. Available from: https://rowman.com/ISBN/9781538172711/Building-Health-Sciences-Library-Collections-A-Handbook.

Developing library collections is an important and difficult task, especially in the health sciences. Collections must suit the needs of their users, but one health science library likely serves multiple different health care professionals and types of students. Users' needs change and information can quickly become outdated in this field. There can also be a disconnect between what librarians think their users want and what users actually want. *Building health sciences library collections: a handbook* covers the basics of collection development and special topics to consider in the health sciences library context. It focuses on providing specific recommendations and defining key topics rather than on guidelines. The editors, Megan Inman and Marlena Rose, both work at the William E. Laupus Health Sciences Library at East Carolina University. Inman is a collection development librarian who serves on the editorial board for the Journal of electronic resources in medical libraries. Rose is the library’s assistant director of Collections and Historical Services (the latter appears to involve archival work). They are well suited as editors based on their current roles and past experiences. The authors of each section are also academic health science librarians who work at universities, making them well-suited to give their contributions; however, it may have been beneficial to include other authors who work at different types of health science libraries.

*Building health sciences library collections* contains a brief introduction by one of the editors about what collection development is, what its components are, and definitions of some key terms used to describe different materials that are common in health sciences libraries. The book is then divided into chapters devoted to special topics in collection development, each written by a contributor. These chapters tend to follow a similar organization of content. The chapters begin with a description of the special topic and why it matters, followed by a series of recommended resources of different types (books, journals, databases, etc.) at the ends of each section where relevant. There is a table of contents at the front of the book as well as an index to help find information about a specific topic. The special topics discussed in this book are as follows: developing diverse and inclusive collections, managing collections through deselection, keeping up to date with emerging topics, developing a medicine collection, developing a nursing collection, developing an allied health collection, open educational resources, and nontraditional online collections (for example, apps).

This book is aimed at a variety of audiences, both newcomers to the profession and those who want to update their knowledge of important and sometimes novel topics in collection development. It is for the most part good at introducing readers to these topics and providing examples of resources that readers might want to acquire. The organization of the book as a whole, including individual chapters and the index, make it easy for someone reading this book for information or resources on a specific topic to quickly find what they need.

There are some notable gaps in the book's coverage though; in the chapter on diversity and inclusivity in collections, resources focusing on Indigenous peoples, intersectionality of different identities, and journals specific to the intersection of health and different areas of marginalization are not discussed at all or not given sufficient examples. All of the contributors are also American, so there may be additional resources that are more suited to a Canadian context that are not considered. As mentioned previously, the contributors are all health science librarians at universities. I believe it would have been valuable to get contributions from health science librarians that work in hospitals, colleges, health centres, or other environments beyond universities. Multiple sections repeated that collections must suit the needs of the users; these different environments may influence needs and user groups. While some of the clinical resources included in certain sections may be useful to these other settings, and most of the recommended resources for colleges would be the same for a university, there might be differences in which resources to prioritize that would be worth distinguishing. This book also could have included a conclusion reflecting on some of the chapters or speculating on other topics or considerations that may be a part of collection development going forward.

The level of detail *in Building health sciences library collections* varies; the introductions of each topic are generally broad and light on specifics, and the recommended resources are rather specific, giving lists of individual titles, databases, etc. that one might want to acquire for a given topic. The writing is very accessible. The authors are able to showcase their points of view in their respective chapters, clearly outlining why each topic discussed is important based on their own experiences and other works. A common theme was the dilemma of health science libraries needing to acquire new and more types of resources in their collections using the same or less funding as they had in the past. As someone who has done some collection development work as a co-op student, I think this book would have been useful before starting some of my projects. It introduced some new resources and concepts that I will keep in mind for future work I do in this area, such as the review services that exist to evaluate different resources.

If one wants an introduction to several important topics in collection development in the health sciences context, this book is a useful purchase. It is most relevant to those in academic health sciences libraries. For the amount of new resources and concepts I learned about, this was worth the price of the book.

